# Multiple large osteolytic lesions in a patient with systemic mastocytosis: a challenging diagnosis

**DOI:** 10.1002/ccr3.1232

**Published:** 2017-10-25

**Authors:** Massimiliano Bonifacio, Roberta Zanotti, Emanuele Guardalben, Elda Mimiola, Francesca Scognamiglio, Omar Perbellini, Giovanna De Matteis, Luis Escribano, Patrizia Bonadonna, Daniela Grigolato, Sergio Bissoli, Alice Parisi, Alberto Zamò, Achille Ambrosetti, Maurizio Rossini

**Affiliations:** ^1^ Department of Medicine Section of Hematology University of Verona Azienda Ospedaliera Universitaria Integrata di Verona Verona Italy; ^2^ Multidisciplinary Outpatients Clinic for Mastocytosis Azienda Ospedaliera Universitaria Integrata di Verona Verona Italy; ^3^ Department of Hematology San Bortolo Hospital Vicenza Italy; ^4^ Department of Life and Reproduction Sciences Section of Clinical Biochemistry University of Verona Verona Italy; ^5^ Servicio Central de Citometria (NUCLEUS) Centro de Investigacion del Cancer (IBMCC; CSIC/USAL) Salamanca Spain; ^6^ Department of Medicine and IBSAL University of Salamanca Salamanca Spain; ^7^ Allergy Unit Azienda Ospedaliera Universitaria Integrata Verona Italy; ^8^ Nuclear Medicine Unit Azienda Ospedaliera Universitaria Integrata di Verona Verona Italy; ^9^ Nuclear Medicine Unit San Giacomo Apostolo Hospital Castelfranco Veneto Italy; ^10^ Department of Diagnostics and Public Health Section of Pathological Anatomy University of Verona Verona Italy; ^11^ Department of Medicine Section of Rheumatology University of Verona Verona Italy

**Keywords:** Non‐hodgkin lymphoma, osteolysis, primary bone lymphoma, systemic mastocytosis, tryptase

## Abstract

Patients with advanced variants of Systemic Mastocytosis may develop destructive bone lesions when massive mast cell (MC) infiltrates are present. Finding of large osteolyses in indolent systemic mastocytosis, typically characterized by low MC burden, should prompt investigations for an alternative explanation.

Systemic Mastocytosis (SM) is a clonal disorder with complex manifestations determined by the proliferation and accumulation of neoplastic mast cells (MC) in the skin and extracutaneous organs (mainly bone marrow, BM) and by the release of soluble mediators. The 2016 revision to the World Health Organization (WHO) classification of mastocytosis includes seven variants: cutaneous mastocytosis, indolent SM (ISM), smoldering SM, SM with an associated hematologic neoplasm (SM‐AHN), aggressive SM (ASM), mast cell leukemia, and mast cell sarcoma 1.

Osteolyses are reported in 5–11% of patients with SM 2, 3, usually associated with osteosclerotic lesions, osteoporosis, or both 4. Small, asymptomatic lesions (<0.5 cm) are reported in about 2% of ISM patients 5, while large osteolyses and pathologic fractures represent one of the “C‐findings” defining ASM 6.

We report about a 55‐year‐old male hospitalized in November 2012 for L2 vertebral fracture and diffuse bone pain needing treatment with opioid analgesics. He had a history of two anaphylactic reactions after bee sting, and he was treated with venom‐specific immunotherapy since 2003. Physical examination performed upon admission was unremarkable, except for neck erythema. He had mild neutrophilia and thrombocytopenia and marked increase in C‐reactive protein and lactate dehydrogenase (Table 1). Of note, serum tryptase was slightly increased (13.2 ng/mL; normal value <11.4 ng/mL). Serum neoplastic markers were all in the normal range. Bone densitometry showed a reduction in T‐score at lumbar spine and femoral neck (−2.2 and −1.0, respectively). CT scan revealed multiple lytic bone lesions in the pelvis (the largest measuring 5 cm), humeri, left scapula, ribs, and vertebral column, without osteosclerotic lesions. Positron emission tomography/computed tomography with 2‐[fluorine‐18]‐fluoro‐2‐deoxy‐D‐glucose (FDG‐PET) demonstrated an increased intensity of FDG uptake in the bone lesions (SUV max 34 in the pelvis). Gastric and colic endoscopies were normal, as well as thyroid evaluation. A BM biopsy was performed, and the major pathological findings were several compact aggregates of CD25^+^/CD117^+^/tryptase^+^ MC, representing around 15% of the cellularity (Fig. 1, upper panel), increased amount of plasma cells (8% of the cellularity with mild increase in lambda monotypic elements), and mild focal fibrosis. Upon these data, the initial diagnosis was ASM with associated monoclonal gammopathy of undetermined significance (MGUS), secondary osteoporosis, and history of anaphylaxis after hymenoptera sting.

**Table 1:** Laboratory data at diagnosis

Parameters	Value	Normal values, range
White blood cell, 10^9^/L	11.87	4.3–10
Neutrophils, %	66.7	43–70
Lymphocytes, %	16.6	25–44
Monocytes, %	9.8	2–12
Eosinophils, %	0.6	0–5
Basophils, %	0	0–2
Platelets, 10^9^/L	121	150–400
Hemoglobin, g/dL	14.3	13.5–17
C‐reactive protein, mg/dL	124	<5
Lactate dehydrogenase, U/L	906	240–480
M‐component IgM/lambda, g/L	3	–
Bence‐Jones proteinuria, mg/L	17	–
Serum calcium, mg/dL	10.2	8.41–10.42
Creatinine, mg/dL	0.7	0.59–1.29
Standard urine examination	Normal	–
Parathyroid hormone, pg/mL	12	6.5–36.8
Vitamin D, ng/mL	25	30–100
Tryptase, ng/mL	13.2	<11.4

**Figure 1:**
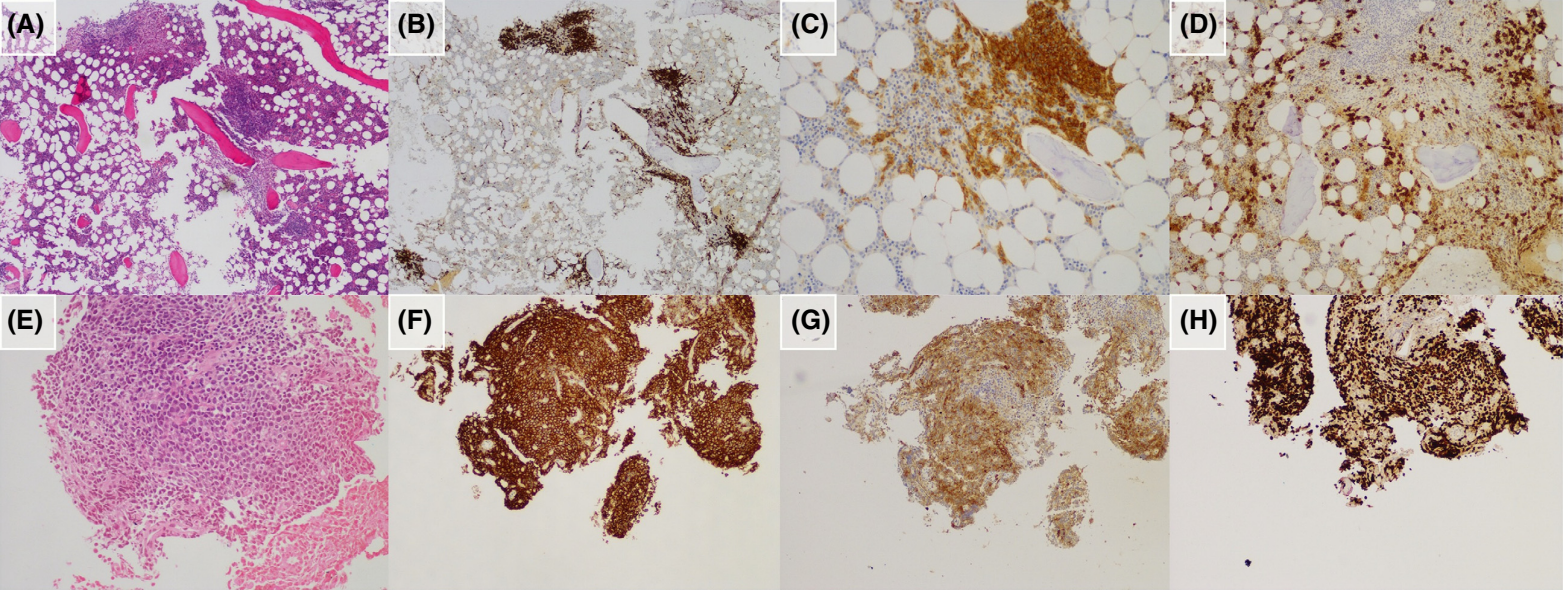
Histopathology and immunohistochemistry of the first bone marrow biopsy (upper panel, A–D) and of the lytic lesion (lower panel, E–H). In the first biopsy, H&E staining shows hypercellular areas (A) that correspond to CD117‐positive mast cell aggregates (B), which show co‐expression of CD25 (C). CD138 (D) shows an increase in the plasma cell population. In the TC‐guided rebiopsy, H&E shows a dense sheet of large cells (E) expressing the B‐cell marker CD20 (F) as well as CD10 (G). The proliferation index as demonstrated by Ki‐67 staining (H) is very high.

The patient was then referred to our Multidisciplinary Outpatients Clinics for Mastocytosis. We repeated the BM aspirate, detecting around 1% MC, 80% of them with spindle‐shape morphology and abnormal distribution of granules (atypical MC type I), corroborating the diagnosis of SM. Multiparametric flow cytometric immunophenotype on BM documented the presence of CD2^+^ and CD25^+^ MC (0.01% of total CD45^+^ cells), and the *KIT* D816V point mutation was detected by ARMS‐qPCR analysis both in BM and in PB 7. Subpopulations of nucleated cells were purified by fluorescence‐activated cell‐sorting (FACS), and the *KIT* mutation was identified in MC, neutrophils, monocytes, and eosinophils, but not in lymphocytes.

The discordance between the low MC burden and the extension of bone involvement prompted us to perform a CT‐guided needle biopsy on a bone lesion of the right ilium, leading to the diagnosis of CD10^+^/BCL6^+^/MUM1^+^/BCL2^+^ diffuse large B‐cell lymphoma (DLBCL) without MC infiltration (Fig. 1, lower panel). The proliferation index, assessed by staining with the monoclonal MIB1 antibody against the Ki67 antigen, was 80–90%. The very small size of the targeted biopsy did not allow the investigation of the *KIT* D816V mutation in that sample. Therefore, the final diagnosis was SM‐AHN: ISM was identifiable as the provisional WHO variant of bone marrow mastocytosis (BMM) 8, and the AHN was classified as the rare entity of primary bone DLBCL 9, 10.

The patient was treated with two cycles of HyperCVAD plus rituximab, obtaining only a partial remission. He then received three cycles of DHAOX plus rituximab, followed by stem cell harvest: after this therapy, FDG‐PET demonstrated complete remission of the lymphoma (Fig. 2), while an iliac crest, nonlesional BM biopsy showed a stable MC infiltration (10% of the BM cellularity). Autologous stem cell transplantation was performed after mitoxantrone and melphalan conditioning in March 2014. Monthly treatment with i.v. zolendronate was administered for 1 year and then every 3–6 months with supplementation of vitamin D for another year. Presently (March 2017), the patient is in continuous complete remission of lymphoma and tryptase levels that are low and stable (3.5 ng/mL). Of note, the specific immunotherapy with Apis venom was withheld at chemotherapy start, with the advice to continue carrying an epinephrine pen injector.

**Figure 2:**
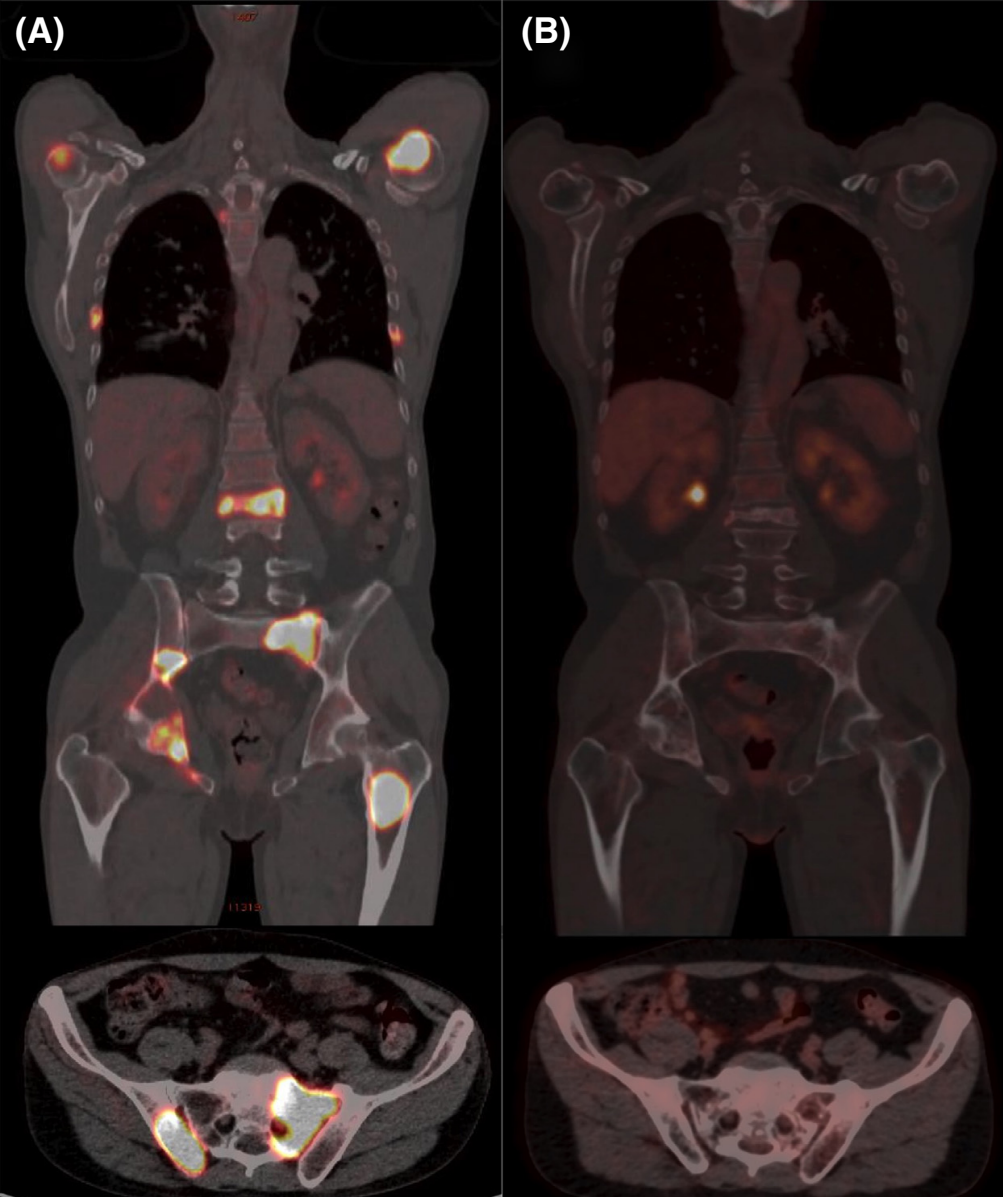
Axial and coronal fused FDG‐PET/CT images in the same patient before treatment (A) showed hypermetabolic bone lesions in both humeri, in some ribs, in a lumbar vertebra, in the pelvis (SUV max 34), and in the left femur. After treatment (B), the FDG uptake in the same areas was normal. Corresponding CT images depicted lytic bone lesions.

Diagnosis of SM can be a challenge in patients presenting without mastocytosis in the skin (MIS). SM without MIS has been historically considered an aggressive disease, with the exception of the BMM variant, an infrequent indolent form characterized by low MC burden with isolated and scattered or multifocal small‐sized atypical MC aggregates and a normal or only slightly increased tryptase level. However, in recent years, it became evident that BMM is more frequent than previously described, particularly in patients presenting with anaphylaxis characterized by hypotension without urticaria/angioedema, or with recurrent mediator‐related symptoms 11, 12. Patients with mastocytosis and anaphylaxis triggered exclusively by insects display significantly different features as compared to other ISM cases, suggesting that they represent a unique subgroup characterized by marked male predominance, low incidence of other MC‐related symptoms, and low serum baseline tryptase levels. Of note, they typically show *KIT* mutation restricted to MC, a distinct feature related to a very good prognosis 13. However, a *KIT* D816V mutation multilineage myeloid and/or lymphoid involvement may occur in these patients, indicating a higher number of mutated hematopoietic progenitors and a higher probability of progression to advanced disease, such as SM‐AHN 14.

The case presented here showed typical characteristics of BMM (history of anaphylaxis after hymenoptera sting, slight increase in tryptase, osteoporosis with vertebral fractures) but the presence of large osteolyses led to a first incorrect diagnosis of ASM. However, at variance with the majority of SM patients presenting with osteolysis, he did not have osteosclerotic lesions. Moreover, the finding of a very high SUV level in bone lesions represented a further element against the hypothesis of ASM. 18‐FDG uptake in SM was evaluated in a series of 19 patients, and pathological BM uptake was documented in nine cases: the median value of SUV max was 4.6 (range 2–12.2) and in 78% of patients the pathologic uptake was associated with diffuse osteosclerosis or a mixed pattern of osteolytic and osteosclerotic lesions at CT scan. Indeed, all these patients were affected by SM‐AHN or MC sarcoma 15.

In conclusion, we suggest that all patients presenting with hypotension/syncope triggered by hymenoptera in the absence of skin symptoms should promptly undergo BM evaluation in order to early identifying a BMM, typically associated with normal or only slightly increased tryptase levels 16. Osteolyses represent a sign of ASM when they are associated with other signs of high MC burden, such as particularly elevated tryptase levels, and when concomitant osteosclerotic lesions are present. In this case, the discordance between the low MC burden and the extensive bone involvement prompted us to perform a direct bone biopsy, allowing the diagnosis of an aggressive lymphoma and avoiding the risk of missing an appropriate intensive chemotherapy treatment, as well as prescribing an unnecessary cytoreduction for mastocytosis.

## Conflict of Interest

All the authors declare no competing interests for this work.

## Authorship

RZ, FS, PB, and MR: performed multidisciplinary clinical evaluation of the patient and collected data. OP, GdM, AP, and AZ: provided flow cytometry, molecular and histological data. DG and SB: provided diagnostic imaging. MB, EG, and EM: wrote the paper. LE and AA: supervised and critically revised the work. All authors approved the final manuscript.
